# Complete Genome Sequence of Streptococcus pneumoniae Strain Rx1, a Hex Mismatch Repair-Deficient Standard Transformation Recipient

**DOI:** 10.1128/MRA.00799-21

**Published:** 2021-10-14

**Authors:** Anna Maria Cuppone, Lorenzo Colombini, Valeria Fox, David Pinzauti, Francesco Santoro, Gianni Pozzi, Francesco Iannelli

**Affiliations:** a Department of Medical Biotechnologies, University of Siena, Siena, Italy; University of Rochester School of Medicine and Dentistry

## Abstract

The complete genome sequence of Streptococcus pneumoniae strain Rx1, a Hex mismatch repair-deficient standard transformation recipient, was obtained by combining Nanopore and Illumina sequencing technologies. The genome consists of a 2.03-Mb circular chromosome, with 2,054 open reading frames and a GC content of 39.72%.

## ANNOUNCEMENT

**FIG 1:**
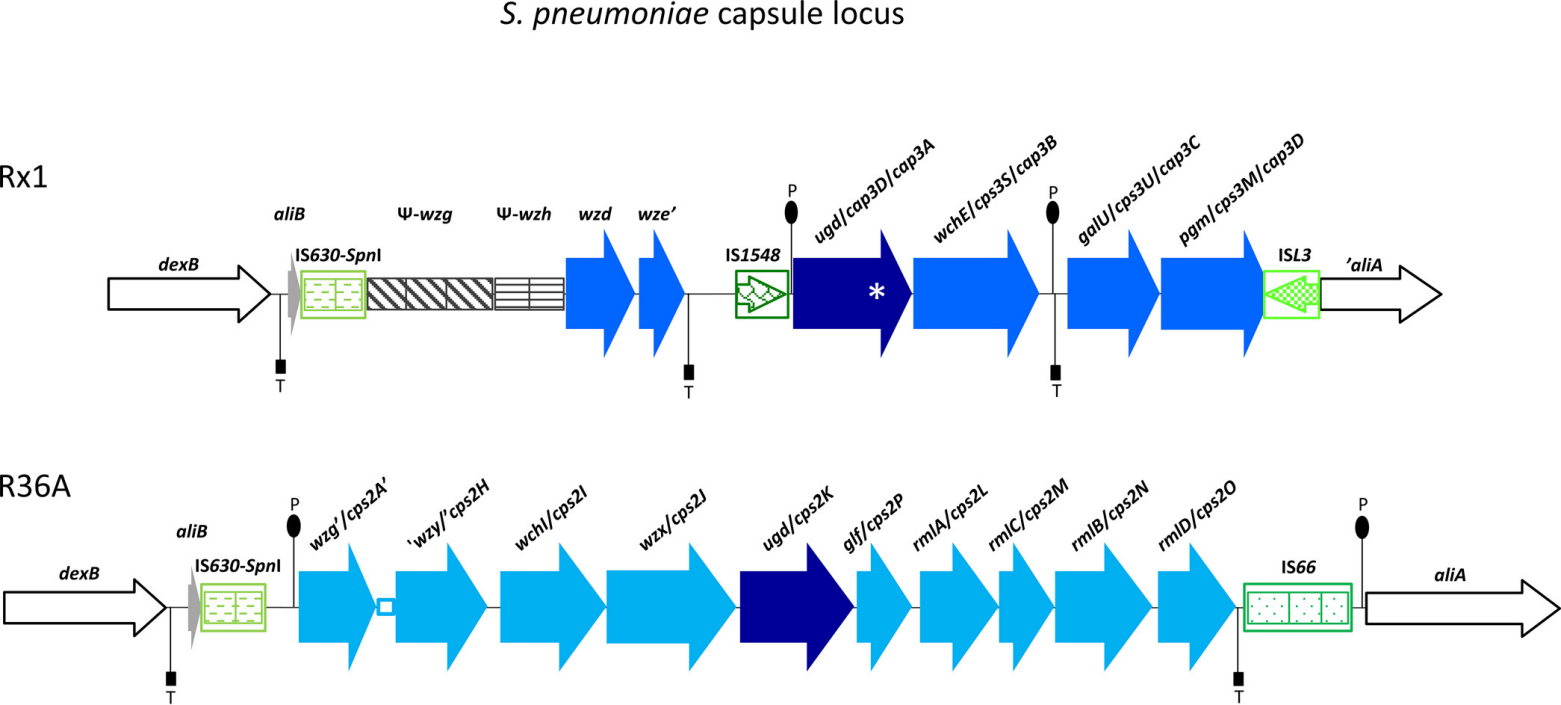
S. pneumoniae capsule locus. Rx1 harbors a type 3 capsule locus acquired by A66 DNA through a double crossover between IS*630*-*SpnI* and *aliA*. At the 3′ end, recombination produced the insertion of an IS*L3* transposase and a 950-bp deletion of the *aliA* 5′ end, as in the A66 capsule locus. IS*1548* identifies (i) a 5′ fragment, common to all serotypes (14), that contains *wzg* and *wzh* pseudogenes and *wzd* and *wze* genes and is not involved in type 3 capsular synthesis (15) and (ii) a 3′ fragment containing *ugd*/*cap3D*/*cap3A* UDP-glucose dehydrogenase gene, *wchE*/*cps3S*/*cap3B* synthase gene, *galU*/*cps3U*/*cap3C*, and *pgm*/*cps3M*/*cap3D* genes involved in UDP-glucose biosynthesis (15–17). The nucleotide change g.317,495C>T in *ugd*/*cps3A*/*cps3D* (indicated with an asterisk) causes p.R320C in the UDP-glucose dehydrogenase UDP-binding domain. The type 2 capsule locus of R36A harbors a 7,505-bp deletion involving the 3′ end of *wzg*/*cps2A*, seven genes (namely, *wzh*/*cps2B*, *wzd*/*cps2C*, *wze*/*cps2D*, *wchA*/*cps2E*, *wchF*/*cps2T*, *wchG*/*cps2F*, and *wchH*/*cps2G*), and the 5′ end of *wzy*/*cps2H* (18). The deletion event left an inverted 25-bp fragment (indicated with an open box) belonging to the lost *wzg*/*cps2A* 3′ end.

Streptococcus pneumoniae is a human pathogen and the most important model organism for studying bacterial genetics and genomics. Widely used laboratory strains include type 2 Avery’s strain D39 and its derivatives Rx1 and R6, which are standard transformation recipients (1, 2). We characterized the complete genome sequence of Rx1, a highly transformable and Hex mismatch repair system-deficient strain. To track the genomic changes that gave rise to Rx1, we also sequenced the genome of its unencapsulated parental strain R36A (Table 1). Strains, which were obtained from the Guild laboratory collection (3), were grown in tryptic soy broth at 37°C for 4 h until they reached an optical density at 590 nm (OD_590_) of 0.8. Pneumococcal cells were harvested by centrifugation (5,000 × *g* for 30 min at 4°C), and the cell pellet was dry vortex-mixed and lysed in 0.1% deoxycholate-0.008% SDS. High-molecular-weight DNA was purified three times with 1 volume of chloroform-isoamyl alcohol (24:1 [vol/vol]), precipitated in 0.6 volumes of ice-cold isopropanol, and spooled on a glass rod. DNA was resuspended in 10× saline-sodium citrate (SSC) buffer (1× SSC is 0.15 M NaCl plus 0.015 M sodium citrate) and then adjusted to 1× SSC and maintained at 4°C. The DNA solution was homogenized using a rotary mixer. Oxford Nanopore Technologies MinION and Illumina HiSeq 2500 instruments were used for DNA sequencing. DNA was not sheared; size selection was obtained with 0.8 volumes of AMPure XP beads (Beckman Coulter). The Nanopore sequencing library was prepared using the SQK-LSK108 kit (Oxford Nanopore Technologies) following the manufacturer’s instructions, and the sample was sequenced using an R9.4 flow cell (FLO-MIN106). Postsequencing high-accuracy base calling and adapter trimming of raw Nanopore reads were performed using Guppy v4.0.11 with configuration dna_r9.4.1_450bps_hac, and base-called reads were analyzed with NanoPlot v1.18.2 (4). Illumina sequencing was performed at MicrobesNG (University of Birmingham) using the Nextera XT library preparation kit (Illumina Inc.), followed by paired-end sequencing. Illumina reads were trimmed using Trimmomatic v0.30 (5) and analyzed with FastQC v0.11.5 (http://www.bioinformatics.babraham.ac.uk/projects/fastqc). Nanopore and Illumina sequencing generated 3,892 long reads (26,780,859 bp [*N*_50_, 18.3 kbp]) and 86,582 read pairs (2 × 250 bp), respectively, for Rx1, whereas 4,771 long reads (27,433,219 bp [*N*_50_, 16.9 kbp]) and 278,462 read pairs were obtained for R36A. Sequence coverage was 31.6× for Rx1 and 67.0× for R36A. A hybrid assembly of Nanopore and Illumina reads was obtained using Unicycler v0.4.712 (6). Assembly completeness and quality were assessed using Bandage v.0.8.1 (7) and Ideel (https://github.com/mw55309/ideel), respectively. Annotation was performed with the NCBI Prokaryotic Genome Annotation Pipeline (PGAP) v5.1 (8). Default parameters were used for all tools unless otherwise specified. The Rx1 genome consists of a 2,030,186-bp single circular chromosome containing 2,054 open reading frames (ORFs), of which 1,813 have a predicted function. The 2,039,955-bp circular chromosome of R36A contains 2,059 ORFs, of which 1,834 have a putative function. Both genomes have (i) a GC content of 39.72%, (ii) 58 tRNA genes, 3 rRNA operons, and 3 structural RNAs, (iii) a 36.6-kb pneumococcal pathogenicity island 1 (PPI1) (9), (iv) prophage remnants, and (v) remnants of the integrative and conjugative element Tn*5253* (10–12). Rx1 and R36A capsule loci are schematized in Fig. 1. Rx1 harbors type I restriction-modification system SpnD39III variant C, while R36A harbors variant D (13). In Rx1, g.168,614C>A, g.1,979,527G>A, and g. 1,629,603delA nucleotide changes introduce premature termination codons in *hexB*, *pspc3.1*, and *dpnC*, respectively.

**TABLE 1:** Genealogy of the S. pneumoniae Rx1 strain

Strain	Description^*a*^	Relevant properties^*b*^	GenBank accession no. (year)^*a*^
D39	Avery’s strain, clinical isolate (1916); type 2, virulent (3, 19–23)	pDP1^+^, Hex^+^, DpnI^+^, *comC1-comD1*, *pspC3.1*	CP000410.1 (2007) (24)
R36	D39 passaged 36 times in anti-type 2 serum (1944); rough, avirulent (3, 21, 22)	pDP1^+^, Hex^+^, DpnI^+^, *comC1-comD1*, *pspC3.1*	Not available
R36A	Highly transformable R36 colony morphology variant (1944); rough, avirulent (3, 20, 23, 25)	pDP1^−^, Hex^+^, DpnI^+^, *comC1-comD1*, *pspC3.1*	CP079922 (2021) (this study)
R6	Highly transformable R36A single-colony isolate (1962); rough, avirulent (3, 26, 27)	pDP1^−^, Hex**^+^**, DpnI^+^, *comC1-comD1*, *pspC3.1*	AE007317.1 (2001) (16)
A66	Avery’s strain, clinical isolate (1949); type 3, virulent (23, 25)	Hex^+^, DpnI, *comC2-comD2*, *pspC11.4*	LN847353.1 (2015) (28)
SIII-N	R36A transformed with A66 DNA (1949); type 3, virulent (20, 23, 25, 29)	*comC1-comD1*, *pspC3.1*	Not available
Rx	Spontaneous rough derivative of R36A (1959); reduced type 3 capsule production, avirulent (3, 17, 23, 30)	pDP1^−^, Hex^−^ (HexB^−^), *comC1-comD1*, *pspC3.1*	Not available
Rx1	Highly transformable derivative of Rx (1959); reduced type 3 capsule production (Ugd mutant), avirulent (3, 31)	pDP1^−^, Hex^−^ (HexB^−^), DpnI^−^ (DpnC^−^), *comC1-comD1*, *pspC3.1*’	CP079923 (2021) (this study)

a The year of the first strain description (except for the D39 isolation year) or of the sequence release is reported in parentheses.

b pDP1 is a 3,161-bp cryptic plasmid (32). Hex is the DNA mismatch repair system encoded by *hexA* and *hexB* (33). DpnI is a restriction system composed of the DpnI/DpnC endonuclease and DpnD (34). *comC-comD* competence genes encode the competence-stimulating peptide (CSP) and its ComD receptor (35–38). *pspC* encodes the virulence surface protein PspC (39, 40).

### Data availability.

The complete genome sequences of R36A and Rx1 are available under GenBank accession no. CP079922 and CP079923, respectively. The sequencing project is available under NCBI BioProject accession no. PRJNA748391. Nanopore and Illumina sequencing reads are available under Sequence Read Archive (SRA) accession no. SRR15216323 and SRR15216322, respectively, for R36A and SRA accession no. SRR15216380 and SRR15216379, respectively, for Rx1.
